# Focus on Opportunities and Adaptive Performance: The Importance of Distinguishing Between Subjective and Objective Performance Measures

**DOI:** 10.1177/00332941241308517

**Published:** 2024-12-16

**Authors:** Lukasz Stasielowicz

**Affiliations:** 9186 Osnabrück University, Osnabrück, Germany

**Keywords:** Future time perspective, focus on opportunities, adaptive performance, adaption to change, performance adaptation

## Abstract

People who perceive more opportunities in their occupational future often report better well-being and motivation. A positive correlation with performance has also been reported, but until now, only the relationship with two broad performance dimensions, task and contextual performance, has been examined extensively. Furthermore, performance ratings often rely on self-reports, which can be biased. The present study (*N* = 258) helps close these research gaps. First, it examines the relationship between focus on opportunities and a specific performance facet (i.e., adaptive performance). Second, performance is assessed both subjectively and objectively. The distinction proved critical. After adjusting for education, focus on opportunities was related to self-reported adaption to change, but there was no substantial evidence that focus on opportunities promotes objective adaptive performance. Thus, it cannot yet be recommended to use focus on opportunities in organizational and educational contexts for selecting and training highly adaptable employees or students.

## Introduction

Future time perspective (Henry et al., 2017) refers to one’s representation of the remaining time in life, such as opportunities, limitations, and perceived remaining time. It has been linked to multiple important variables. According to meta-analyses, correlates of future time perspective include achievement, health behavior, well-being, and risk behavior (Andre et al., 2018; Diaconu-Gherasim et al., 2021; Kooij et al., 2018). Such relationships could have implications for organizations and educational institutions. For example, better employee well-being, less risky behavior, and better performance could improve organizational efficiency and reduce costs. Thus, it is unsurprising that the importance of future time perspective was also investigated in applied contexts, such as organizations. In one meta-analysis, people’s perception of their own occupational future time (e.g., opportunities and limitations) was related to several organizational variables, including well-being, motivation, and performance (Rudolph et al., 2018). However, the strength of the relationship differed between various future time perspective dimensions. Therefore, the present study will focus on the dimension that displayed the strongest relationships in the past.

Rudolph and colleagues (2018) distinguished three possibilities for measuring future time perspective: overall measure, focus on opportunities, and perceived remaining time. The latter two are considered different dimensions of future time perspective (Cate & John, 2007; Zacher & Frese, 2009). Thus, different correlation patterns with potential outcomes, such as performance, are possible. Indeed, in the mentioned meta-analysis (Rudolph et al., 2018), the highest mean correlations with performance were found for focus on opportunities rather than perceived remaining time. Specifically, a moderate correlation was identified with contextual performance (i.e., voluntary employee activities contributing to the organization) and a small mean correlation with task performance. However, a more nuanced investigation of the relation between focus on opportunities and job performance is needed, as the general task performance consists of multiple dimensions (Campbell & Wiernik, 2015). Currently, it is unclear to what extent organizations and educational institutions should account for focus on opportunities when developing interventions to optimize employees’ or students’ performance levels and to what extent focus on opportunities should be considered in personnel selection or student admission tests, where specific performance dimensions are critical. This uncertainty can be partially attributed to the reliance on self-report performance measures, which are not necessarily valid performance indicators (Heidemeier & Moser, 2009). To help close this research gap, the present study examines the relationship between focus on opportunities and both subjective and objective measures of a specific performance dimension - adaptive performance.

### Adaptive Performance

Adaptive performance refers to “altering behavior to meet the demands of a new situation, event, or set of circumstances” (Pulakos et al., 2000), and researchers have shown that it can be conceptually and empirically differentiated from other performance constructs, such as task proficiency and task proactivity (M. A. Griffin et al., 2007). Since many modern work environments are very dynamic (e.g., new technologies and changing team size), employees and students must be able to adapt to changes (Bohle Carbonell et al., 2014; Ryan & Ployhart, 2014). Thus, it is not surprising that there was a surge of studies examining adaptive performance at the beginning of the 21^st^ century. Following the surge, a few research groups summarized the available findings, including plausible antecedences of individual adaptive performance (Baard et al., 2014; Jundt et al., 2015). Some of the correlates were also examined in quantitative meta-analyses. Specifically, personality factors (Huang et al., 2014; Woo et al., 2014), goal orientation (Stasielowicz, 2019), and cognitive ability (Stasielowicz, 2020) were investigated. Although non-negligible mean correlations were found in all of the mentioned meta-analyses, the latter two systematic reviews found that the strength of the relationship depends on how adaptive performance is measured. Therefore, the role of the measurement method is also considered in the present study.

Similar to other fields, researchers examining adaptive performance distinguish between subjective and objective measures of adaptive performance (Christian et al., 2017; Stasielowicz, 2019, 2020). Subjective ratings are usually collected by asking people to rate their adaptive performance or the performance of others, for example, co-workers or employees (Bohle Carbonell et al., 2014; Stasielowicz, 2022; Stokes et al., 2010). In contrast, people are asked to complete a specific task to measure adaptive performance objectively. Tasks used in previous studies include radar-tracing simulations (Kozlowski et al., 2001), video games (Hardy et al., 2014), and creating presentation slides (Keith et al., 2010). Participants are asked to complete a task, but the requirements change after a certain period or a pre-determined number of trials. Performance indicators such as accuracy, speed, or fulfilling specific criteria are then used to capture adaption to change.

Since task scores are often automatically determined by computers, relying on them can be more advantageous than when using ratings provided by people. However, completing tasks usually requires more time than providing subjective ratings. Sometimes, completing the tasks can take several hours (Keith et al., 2010; Kozlowski et al., 2001). Furthermore, a specific task cannot simultaneously assess all kinds of adaptive performance. In contrast, questionnaires that collect subjective ratings usually distinguish between several adaptive performance dimensions. Initially, eight adaptive performance dimensions were proposed (Pulakos et al., 2000): (1) solving problems creatively, (2) dealing with uncertain or unpredictable work situations, (3) learning new tasks, technologies, and procedures, (4) demonstrating interpersonal adaptability, (5) demonstrating cultural adaptability, (6) demonstrating physically oriented adaptability, (7) handling work stress, and (8) handling emergencies or crisis situations. In further research projects, some dimensions were occasionally dropped because they were irrelevant to some jobs (Stokes et al., 2010). To illustrate, physical adaptive performance is more relevant in the military context than in the office.

While the eight-dimensional taxonomy proposed by Pulakos and colleagues (2000) was often used as a starting point in subsequent research, more parsimonious taxonomies were proposed in various studies. One questionnaire differentiates between five dimensions (Charbonnier-Voirin & Roussel, 2012): (1) creativity, (2) reactivity in the face of emergencies or unexpected circumstances, (3) interpersonal adaptability, (4) training and learning effort, and (5) managing work stress. In another questionnaire, the original eight-dimensional taxonomy was collapsed into two dimensions (Kröger & Staufenbiel, 2012): social adaptive performance and task-oriented adaptive performance. Social adaptive performance encompasses interpersonal and intercultural adaptive performance dimensions, and task-oriented adaptive performance is based on the remaining original dimensions, except physically oriented adaptive performance. The collected empirical evidence supported the two-dimensional structure to a greater extent than structures with more dimensions. Therefore, this two-dimensional distinction will also be considered in the present study.

The main goal of the present study is to examine the relationship between adaptive performance and the dimension of future time perspective, which showed the strongest meta-analytical relationship with other performance facets - focus on opportunities (Rudolph et al., 2018). The current study goes beyond state-of-the-art research by assessing performance both subjectively and objectively, which will provide a more comprehensive perspective than studies relying on a single performance measure. This decision is motivated by the fact that substantially different correlations with other variables were identified in meta-analyses examining subjective adaptive performance ratings and objective adaptive performance scores (Christian et al., 2017; Stasielowicz, 2019, 2020). Furthermore, including objective performance measures addresses the problem of limited validity of self-report measures (Heidemeier & Moser, 2009; Mabe & West, 1982), which studies examining the relationship between focus on opportunities and performance are usually based on. Thus, the present study contributes to solving the problem of the limited utility of findings for organizational and educational processes.

In the current study, subjective ratings will be collected using the two-dimensional taxonomy differentiating between social and task-oriented adaptive performance (Kröger & Staufenbiel, 2012). In addition, objective scores will be registered using a stock-pricing task. The expected relationship between focus on occupational opportunities and adaptive performance is described in the next section.

### Focus on Opportunities and Adaptive Performance

Focus on occupational opportunities is regarded as one dimension of the occupational future time perspective. While future time perspective refers to one’s perception of remaining time and opportunities in life in general, occupational future time perspective focuses on the job context (Henry et al., 2017), with which the present study is also primarily concerned. Although many researchers thought of future time perspective as a single dimension, more recent work established that distinguishing between focus on opportunities and focus on limitations or perceived remaining time is important (Cate & John, 2007; Mohammed & Marhefka, 2020; Zacher, 2013). Cate and John (2007) provided evidence that the levels of the different dimensions change at a different rate with age. Age-related differences were also found in the occupational context (Weikamp & Göritz, 2015).

As mentioned in the introduction, the future time perspective has been linked to better achievement, health behavior, and well-being (Diaconu-Gherasim et al., 2021; Kooij et al., 2018). While such relationships might be relevant to processes in the organizational or educational context, it is vital to determine whether future time perspective affects performance before recommending considering the future time perspective in personnel selection, student admission tests, and employee as well as student training. Unfortunately, the relationship with performance was only extensively examined for a few performance facets (Rudolph et al., 2018). On average, focus on opportunities, rather than perceived remaining time or overall future time perspective, showed the strongest relationship with task and contextual performance. Since that dimension of future time perspective is most promising, focus on opportunities will also be considered in the present study examining the relationship with adaptive performance – a performance dimension that is crucial in modern dynamic work and educational environments (Bohle Carbonell et al., 2014; Ryan & Ployhart, 2014).

Hitherto, the relationship with adaptive performance was largely neglected. The importance of focus on opportunities was not yet investigated. However, a few studies examined the relationship between the overall future time perspective and adaptive performance (Abrantes et al., 2020; Shaw et al., 2019). In one study, future orientation was positively related to self-reported team adaptation and performance on one of two dynamic tasks (Abrantes et al., 2020). Another study reported a small positive correlation between future time perspective and individual adaptive performance on a test battery (Shaw et al., 2019). Thus, there is a lack of studies (a) using both subjective and objective adaptive performance measures at the individual rather than team level and (b) examining the relationship with focus on opportunities rather than overall future time perspective. Concerning the former, differentiating between subjective and objective adaptive performance measures is important because the magnitude of their relationship with other variables often varies considerably according to meta-analytic findings (Christian et al., 2017; Stasielowicz, 2019, 2020). For example, the relationship between goal orientation and adaptive performance is stronger when using subjective rather than objective measures of adaptive performance. As to the latter point, examining the relationship with focus on opportunities rather than overall future time perspective or perceived remaining time could be fruitful because the focus on opportunities showed the strongest relationship with other performance facets (i.e., task and contextual performance) in one meta-analysis descriptively (Rudolph et al., 2018). The present study aims to close the mentioned research gaps by examining the relationship between focus on opportunities and both subjective and objective adaptive performance at the individual level. For the reasons described in the following paragraphs, a positive relationship between focus on opportunities and adaptive performance is expected in the present study.

According to one theoretical framework (Jundt & Shoss, 2023), the adaption process can be influenced by various aspects, such as detecting or anticipating change, identifying change-related demands, choosing and implementing strategies to deal with change, and revising strategies through learning. As Jundt and Shoss note in their theoretical article (2023) and empirical studies based on longitudinal modeling show (Richels et al., 2020; Stasielowicz, 2024), various emotional, cognitive, and motivational variables can promote or impair the adaption process at different stages. A theoretical link between focus on opportunities and adaptive performance can be made through self-regulatory mechanisms. Occupational focus on opportunities is usually operationalized as positive thinking about occupational opportunities (Zacher et al., 2010), and this positive thinking could help set goals conducive to good performance. After all, according to meta-analytic findings, future time perspective is related to greater goal setting (Baird et al., 2021), which can help simulate and plan activities, resulting in better performance (Rudolph et al., 2018; Zacher et al., 2010). Furthermore, future time perspective is associated with goal monitoring, goal operating, and self-regulatory ability (Baird et al., 2021), which might result in greater persistence, concentration, and better time management (de Bilde et al., 2011), and be beneficial in the performance context.

Through the lens of the adaptive performance model (Jundt & Shoss, 2023), focus on opportunities might be relevant at different stages of the adaption process. People generally perceiving more opportunities might be more likely to anticipate or detect changes because such events might constitute additional opportunities. Detecting changes early might promote adaptive performance, as people might realize faster that current routines are not effective anymore. Such insights might prompt people to stop relying on automatic processes and instead devote attentional resources to examining the changed environment, selecting new strategies, and monitoring their implementation. Thus, focus on opportunities might speed up the adaption process, reducing organizational losses.

Available empirical findings are consistent with the presented theoretical framework of the adaption process. While the specific relationship between focus on opportunities and individual adaptive performance has not been examined yet, there is some evidence that the overall future time perspective is related to adaptive performance (Abrantes et al., 2020; Shaw et al., 2019). Future-oriented people might be interested in understanding how the current situation could impact the future (Shaw et al., 2019). Since unexpected changes in work environments can result in performance impairment, thinking about future consequences of disruptions could help people recognize the need to show adaptive performance (Abrantes et al., 2020). While these findings are helpful, several research gaps remain; the relationship between focus on opportunities, a specific dimension of future time perspective, and adaptive performance has not been examined at the individual level.

Arguably, seeing more opportunities could be helpful when adapting to changes. When faced with a changing environment and receiving negative feedback, focus on opportunities could prompt people to evaluate the difference between the current state and future goals. Abrantes and colleagues (2020) point out that future-oriented people face a dilemma in dynamic situations. While executing untested actions can compromise future goals, taking action is necessary to prevent endangering future goals. Given that people often show action bias in decision-making (Patt & Zeckhauser, 2000), one could speculate that people focusing on opportunities will be more willing to actively prevent the negative consequences of bad performance rather than risk facing bad outcomes through inaction. Realizing a discrepancy between the current state and their goals could motivate people to change their behavior. For example, people may search for solutions more quickly or put more effort into finding solutions, leading to better adaptive performance.

Based on the mentioned findings and theoretical plausibility, a positive relationship between focus on opportunities and adaptive performance is expected in the present study. Both subjective ratings and objective scores will be used to gauge adaptive performance. Subjective ratings of adaptive performance will differentiate between social adaptive performance (e.g., dealing with new colleagues or clients from foreign cultures) and task-oriented adaptive performance (e.g., learning new procedures or dealing with emergencies). This distinction between different kinds of subjective ratings enables examining whether the focus on opportunities is related to different types of performance to a similar extent. Theoretically, focus on opportunities could help detect changes and set goals in various areas. Thus, it could be related to both social and task-oriented adaptive performance.

  Hypothesis 1: There is a positive linear relationship between focus on opportunities and self-reported social adaptive performance.

  Hypothesis 2: There is a positive linear relationship between focus on opportunities and self-reported task-oriented adaptive performance.

Objective adaptive performance scores in the present study are based on a task that changes after a certain number of stimuli are presented. Since computing the mean post-change performance does not capture the adaption process, longitudinal modeling of adaptive performance has been proposed (Bliese & Lang, 2016). It allows one to gauge *transition adaption*, which reflects performance impairment observed immediately after the change. Better transition adaption is reflected by smaller performance impairment. Another relevant concept is *reacquisition adaption*. It is based on the (re-)learning process in the changing environment after the initial performance impairment. Fast (re-)learning after the change-induced performance impairment can be regarded as an indicator of successful adaption.

A closer look at the process model of adaptive performance (Jundt & Shoss, 2023) makes it clear that both transition and reacquisition could theoretically benefit from a focus on opportunities. A stronger focus on opportunities might be beneficial in the early stages of the adaption process, which comprises anticipating and detecting change. Identifying a discrepancy directly after the change between the current performance level and one’s goals could prompt people to modify their behavior faster and find better strategies, resulting in better transition adaption. As evidenced by the relationship between focus on opportunities, goal setting, goal monitoring, concentration, and time management (Baird et al., 2021; de Bilde et al., 2011), the benefits of focus on opportunities could also extend into later stages of the adaption process, resulting in greater improvements (i.e., reacquisition adaption) than in people with a relatively weak focus on opportunities.

  Hypothesis 3: Focus on opportunities promotes transition adaption during the change task.

  Hypothesis 4: Focus on opportunities promotes reacquisition adaption during the change task.

Notably, the present study accounts for potential confounding across all four hypothesis tests. Variables related to both focus on opportunities and adaptive performance need to be considered to provide accurate effect estimates. Since demographic variables usually causally precede other variables, they are good candidates for the adjustment set. While age and sex are not consistently related to adaptive performance (Jundt et al., 2015), education is related positively to both focus on opportunity and adaptive performance (Jundt et al., 2015; Rudolph et al., 2018). Therefore, the present study adjusts for education by including it as a predictor in regression models (Cinelli et al., 2022).

## Methods

### Design

Data collection followed a two-step approach. First, the data from a convenience sample were collected online between February 2018 and July 2018. Second, a panel sample was recruited from the Clickworker platform in January 2019 to reach as many occupationally active people as possible. The convenience sample was included because both focus on opportunities and adaptive performance are relevant to other applied contexts, such as education (Andre et al., 2018; Bohle Carbonell et al., 2014). All study materials were presented in German. In general, it took approximately 30 minutes to complete the survey. In total, 528 people answered at least one question.

### Procedure

Following a short introduction, participants provided informed consent in the online survey. Next, demographic information was collected. Only people over 18 years old received additional questions: job complexity, learning goal orientation, avoid performance goal orientation, focus on opportunities, and adaptive performance questionnaires. Some of these questions were used for undergraduate projects and are not considered in this study.

Next, participants were asked to complete a task to measure adaptive performance objectively. Participants were asked to imagine that they were new employees in a bank. They were told their job was to recommend stock acquisitions to clients. The task was introduced as a part of a training program. Specifically, participants were asked to assess the potential (0 = low, 30 = high) of different stocks by using two indicators: company rating provided by a professional rating agency (1 = low creditworthiness, 4 = very high creditworthiness), and profit in the previous year (1 = ca. 10% of the revenue, 4 = ca. 40% of the revenue). The 16 possible combinations of indicators formed one set of stimuli. Eight sets (128 stimuli) were used in total, with four sets presented in the pre-change phase and four in the post-change phase.

**Figure 1.**
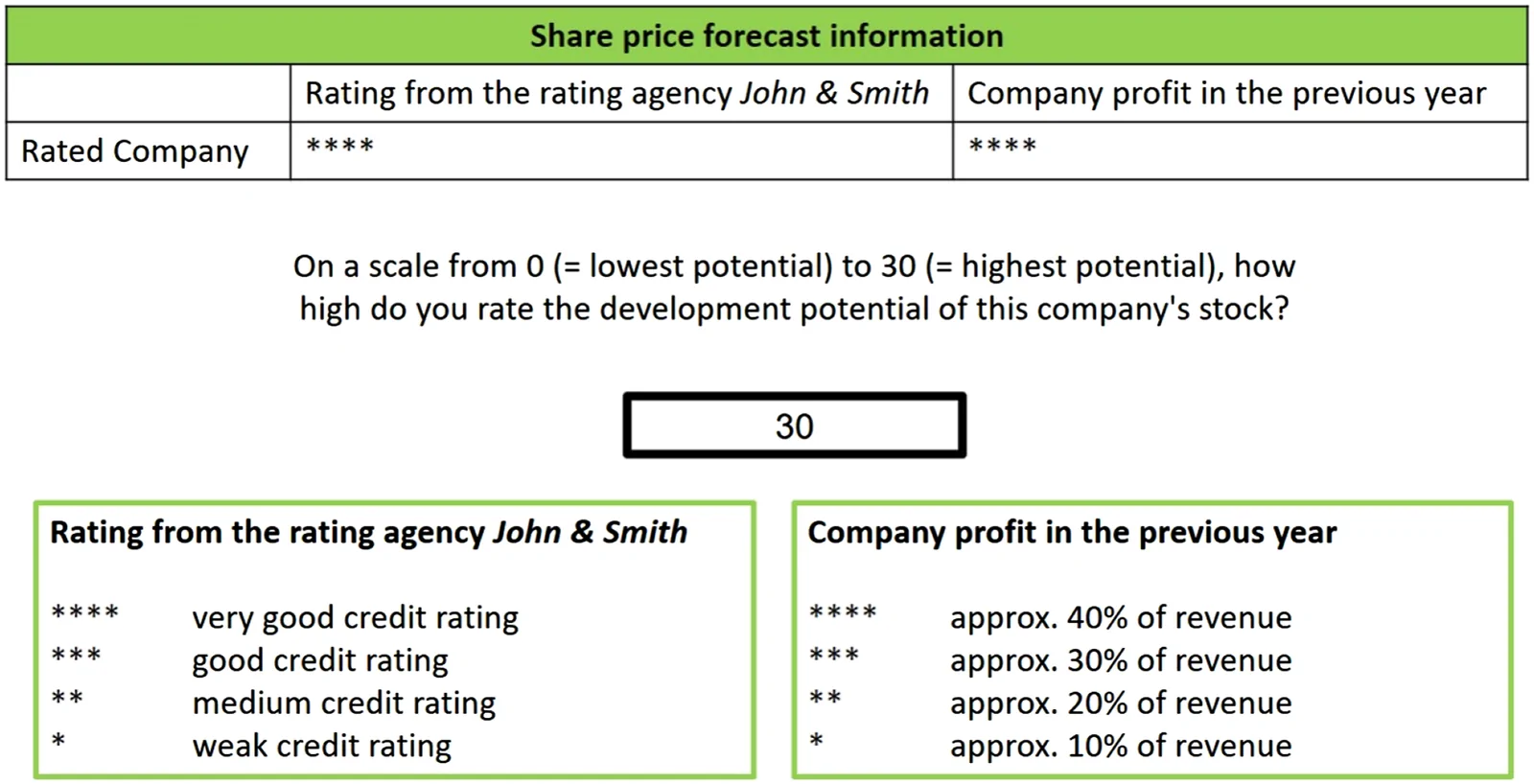
Example item of the share price forecast task used to measure adaptive performance (translated from German). *Note*. Participants were asked to rate (0–30) the development potential based on two indicators: rating from a professional rating agency and company profit in the previous year. To measure adaptive performance, the importance of the indicators changed during the task. In the pre-change phase, company profit was more important than the rating agency’s opinion. In contrast, the rating agency’s opinion was more important in the post-change phase.

The task rules were explained by showing an example item with maximal values of both indicators (see Figure 1). The participants were informed that 30 (= highest share price potential) would be the correct response in this scenario and were asked to submit this value. Depending on the answer, participants received feedback on their screen on whether their rating was accurate. In addition, the expected answer was shown. They were also informed that the reliability of the indicators could change due to certain events, such as a stock market crash.

The participants were given four sets of trials to master the task. Initially, the profit indicator was more important than the creditworthiness indicator. Although the underlying equation was not disclosed, participants could use the feedback about the response accuracy to optimize their performance. After completing the fourth block, the indicator weights changed without informing the participants. However, participants still received feedback, which they could use to improve their performance.

After completing the eight task blocks, each participant in the panel sample received 5 € for completing the study. Participants from the convenience sample could choose between a course credit and a draw (four 20 € vouchers) or reject compensation.

### Measures

#### Focus on Opportunities

Three items (α = .84, 95% CR [.81, .88]) were used to assess focus on opportunities using a five-point scale (1 = does not apply at all, 5 = applies completely). An example item is “My occupational future is filled with possibilities” (Zacher et al., 2010).

#### Subjective Adaptive Performance Ratings

Self-reported social and task-oriented adaptive performance were assessed by a scale consisting of 18 items (Kröger & Staufenbiel, 2012). In contrast to other adaptive performance questionnaires, the scale developed by Kröger and Staufenbiel was extensively validated in German samples. Six items (α = .78, 95% CR [.74, .82]) assess social adaptive performance, for example, “I quickly establish contacts with strangers (e.g., new colleagues, clients).”. The remaining 12 items gauge task-oriented adaptive performance (α = .82, 95% CR [.79, .85]), for example, “I make well-thought-out and goal-oriented decisions in emergencies.” Across all items, people are asked to rate their performance on a seven-point scale (1 = I do not agree at all, 7 = I agree completely).

#### Objective Adaptive Performance Scores

Following previous studies (Howe, 2019; Lang & Bliese, 2009), a task was used to measure adaptive performance objectively. Specifically, the stock task described in the previous section was used. Participants were asked to estimate the share price potential (0–30) based on the two indicators (i.e., company profit and creditworthiness rating). Crucially, the task requirements changed without direct warning after a certain number of trials, enabling measuring adaption to change. In the pre-change phase, the profit indicator was more important than the rating agency’s opinion. Specifically, the share price potential could be estimated using the following formula, which was not disclosed to the participants:

Share price potential = 4*Agency_Rating + 6*Company_Profit – 10.

To illustrate, an agency rating of 3 and a company profit of 2 corresponded to a share price potential of 14 (4*3 + 6*2 – 10).

In the post-change phase, the rating agency’s opinion was more important than the company’s profit. Specifically, the underlying formula was changed to:

Share price potential = 6*Agency_Rating + 4*Company_Profit – 10.

Accordingly, an agency rating of 3 and a company profit of 2 corresponded to a share price of 16 (6*3 + 4*2 – 10).

Based on the answers, the performance was computed. Specifically, participants’ estimates of share price potential were subtracted from the actual potential. Since both overestimation and underestimation were regarded as errors, absolute values of the difference were recorded. The differences were then averaged across the set of 16 stimuli for each of the eight blocks. The best possible score in each block was 0, which would imply that the participant’s response was identical to the expected answer (i.e., real share price potential) on all 16 trials of one block. The worst possible score was 21.75, implying that the participant’s estimate of share price potential was always the furthest from the actual potential.

### Statistical Analyses

Primary analyses were conducted using the R package brms for Bayesian modeling. Bayesian estimation (Kruschke & Liddell, 2017) uses prior knowledge (e.g., negative variances are impossible, huge effect sizes are unlikely) and data of the participants (likelihood) to estimate the parameters of interest (e.g., correlations or regression coefficients). The resulting distribution of the estimates (i.e., posterior distribution) is based on the Markov Chain Monte Carlo (MCMC) method. Candidate values (e.g., correlation = .75) are either accepted or rejected based on the priors and available data. The accepted values build a chain of values. However, the first chain values are usually discarded because the starting point is often chosen randomly, which means that it may take some time before the algorithm starts exploring plausible values (warm-up phase). Furthermore, several independent chains are recommended to enable identifying potential problems, such as different chains exploring different regions. The undiscarded values of all chains are then used to describe the posterior distribution – the most plausible results, given the prior knowledge and available data. This distribution can be used to quantify the uncertainty of the estimates (Morey et al., 2016), such as the limits of a credibility interval containing a certain percentage of the most plausible values (e.g., 95% CR).

In the present study, weakly informative priors are used, allocating more prior probability to smaller rather than larger correlations or regression coefficients. This decision is motivated by the fact that effect sizes reported in the organizational literature are rarely large (Bosco et al., 2015). Each analysis is based on four Markov chains with 2000 post-warm-up iterations. Thus, each posterior distribution is estimated using 8000 samples.

#### Subjective Adaptive Performance Ratings

Regression models will be used to examine the relationship between focus on opportunities and subjective adaptive performance ratings. Specifically, focus on opportunities and education (i.e., a potential confounder) will be included as predictors of self-reported adaptive performance. Separate models will be built for self-reported social adaptive performance and self-reported task-oriented adaptive performance.

#### Objective Adaptive Performance Ratings

A framework developed by Lang and Bliese will be used to model the performance trajectories in the change task. Hitherto, the framework was applied in multiple studies (Howe, 2019; Lang & Bliese, 2009; Niessen & Jimmieson, 2015; Niessen & Lang, 2021), and it involves using several change variables to model performance across time. The values of the change variables across the eight measurement occasions are fixed according to the principles recommended for studies examining transitions (Bliese & Lang, 2016). Table 1 contains the values of the change variables at specific measurement occasions, and their meaning is described in the next paragraph.

**Table 1.** Coding of Change variables Capturing adaptive performance Trajectories. See text for Explanations.

	Measurement occasion							
	Pre-change phase				Post-change phase			
Change variable	1	2	3	4	5	6	7	8
SA (skill acquisition)	0	1	2	3	4	5	6	7
SA^2^ (quadratic SA)	0	1	4	9	9	9	9	9
TA (transition adaption)	0	0	0	0	1	1	1	1
RA (reacquisition adaption)	0	0	0	0	0	1	2	3
RA^2^ (quadratic RA)	0	0	0	0	0	1	4	9

The chosen coding scheme ensures that the estimated parameter values can be used to describe the average performance trajectory. Specifically, skill acquisition (SA) refers to the learning rate in the task. The SA value of zero at the first measurement occasion (see Table 1) implies that the intercept in the regression model corresponds to the average performance on the first measurement occasion. Since the learning rate can change during the task (e.g., considerable improvements at the beginning and minor improvements in the later trials), not only a linear trend (SA) but also a quadratic trend for skill acquisition (SA2) is considered in the present study. Furthermore, the sudden change of task requirements introduced after the fourth measurement occasion is expected to result in immediate performance impairment, which is captured by another change variable: transition adaption (TA). It corresponds to the difference between the actual and expected performance had there been no change (i.e., prolonged skill acquisition in the pre-change phase). Finally, reacquisition adaption (RA) refers to the difference between the pre- and post-change learning trends. RA and RA2 of zero would mean that the performance in the post-change phase improved as quickly as in the pre-change phase.

Interaction terms involving change variables (e.g., TA) are used to examine the relevance of focus on opportunities. If focus on opportunities facilitates adaptive performance, one would expect a negative sign of the interactions involving TA and RA terms. A negative sign would mean that people with a stronger focus on opportunities show a smaller performance decrease directly after the change (TA) and a greater improvement rate in the later trials (RA). Including focus on opportunities in the model slightly modifies the meaning of the simple change parameters. Since focus on opportunities was centered on the sample mean, the change variables’ coefficients in the final model describe the average performance of people with an average focus on opportunities.

**Figure 2.**
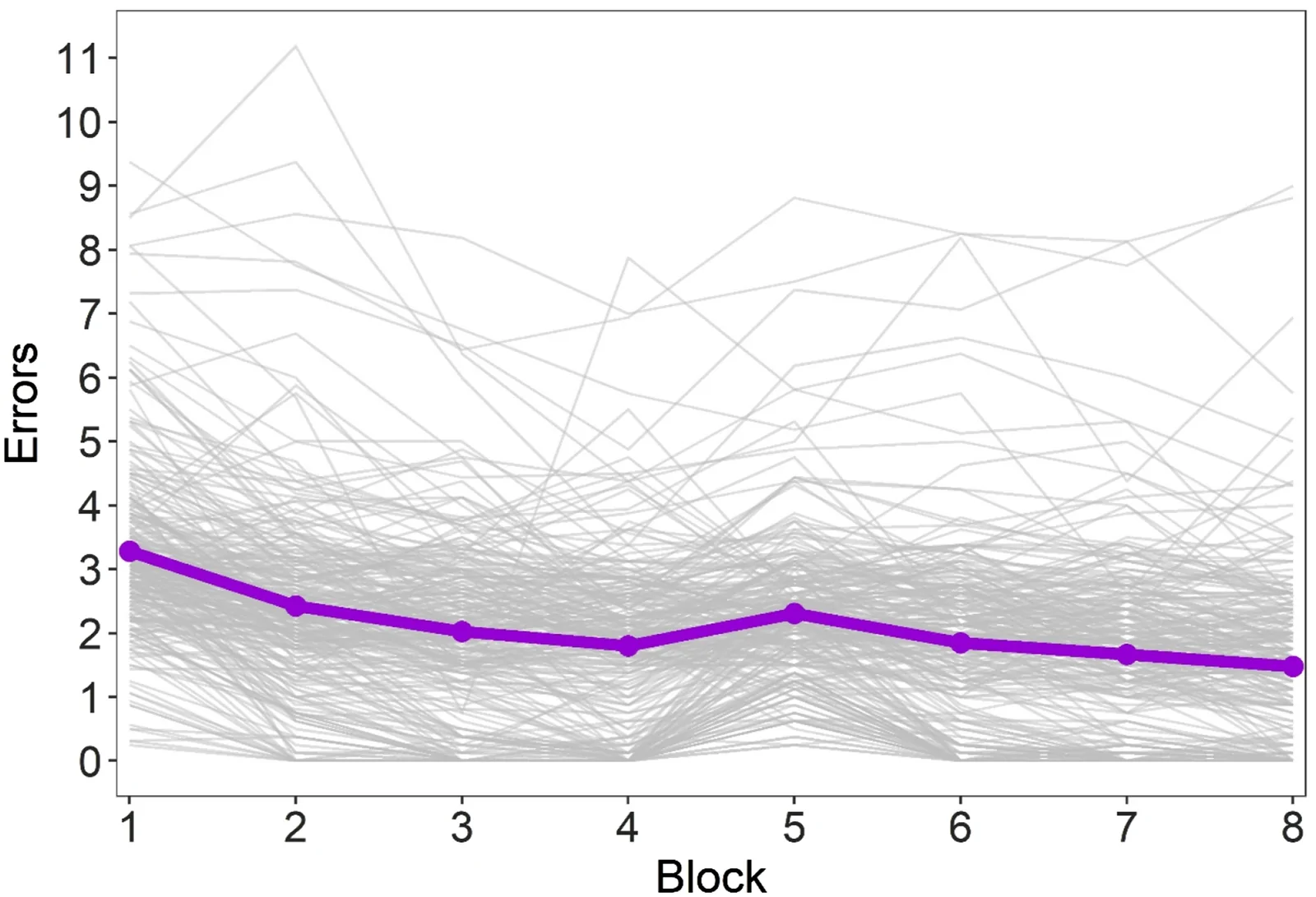
Performance trajectories before the change (blocks 1–4) and after the change (blocks 5–8). The wide violet curve reflects the average performance on the task, and the thin grey curves are individual trajectories (*N* = 258).

Data dependency (i.e., several measurement occasions per person) is accounted for using multi-level models. The performance on the eight measurement occasions (level 1) is nested in individuals (level 2). Multi-level models also allow one to account for the fact that individual performance trajectories may deviate from the average performance curve; for example, participants might have different starting points. Thus, some deviations reflect real differences between people rather than measurement error. Visual inspection of individual trajectories (Figure 2) confirmed substantial variability between performance curves in the present study. Therefore, linear mixed-effects models will be used in the present study, which include fixed effects to describe the average performance trajectory and random effects to describe real deviations from the average.

In the first step, a model reflecting performance trajectories over the eight measurement occasions was built. Fixed effects were included for all linear and quadratic change variables, such as SA2 and TA. Random effects were also included for all change variables. Different starting points of the trajectories were also allowed (random intercept). Next, the relationship between focus on opportunities and objective adaptive performance scores was examined. Fixed effects were added for focus on opportunities (relationship with the intercept) and interactions involving focus on opportunities and all change variables. The interactions of focus on opportunities with TA and RA are particularly interesting in the present study as they capture the adaption process. The model also included education and its interactions with the change variables to account for confounding.

#### Transparency and Openness

The study and the analyses were not pre-registered. Data, all prior specifications, a list of packages, R code, and output are available at https://osf.io/2yeg3.

## Results

### Sample

The final sample consisted of 258 people. Of 528 participants who answered at least one question in the online survey, 217 did not complete the adaptive performance task and had to be excluded. Furthermore, after closer inspection, data from some of the remaining 311 people who completed the study had to be excluded. Three people provided the same answer on the adaptive performance task for all 16 task stimuli in more than two of the eight stimuli blocks, indicating that they were unwilling or unable to learn the task.

Furthermore, 49 people who did not show skill acquisition in the pre-change phase were excluded; the performance at the end of the pre-change phase (fourth block) was worse than at the beginning of the task (first block). Only one participant was excluded due to other criteria. Specifically, one person provided the same answer to more than four out of seven questionnaires (six) and completed the study faster than 95% of the sample. It was the fastest participant (8.23 minutes), and the speed was only slightly below the plausible physiological threshold of approximately 1000 words per minute. Thus, the data from 258 participants could be considered in the main analyses. As such, the present study was less plagued by inattentive responses than other online studies (Ternovski & Orr, 2022).

### Descriptive Statistics

**Figure 3.**
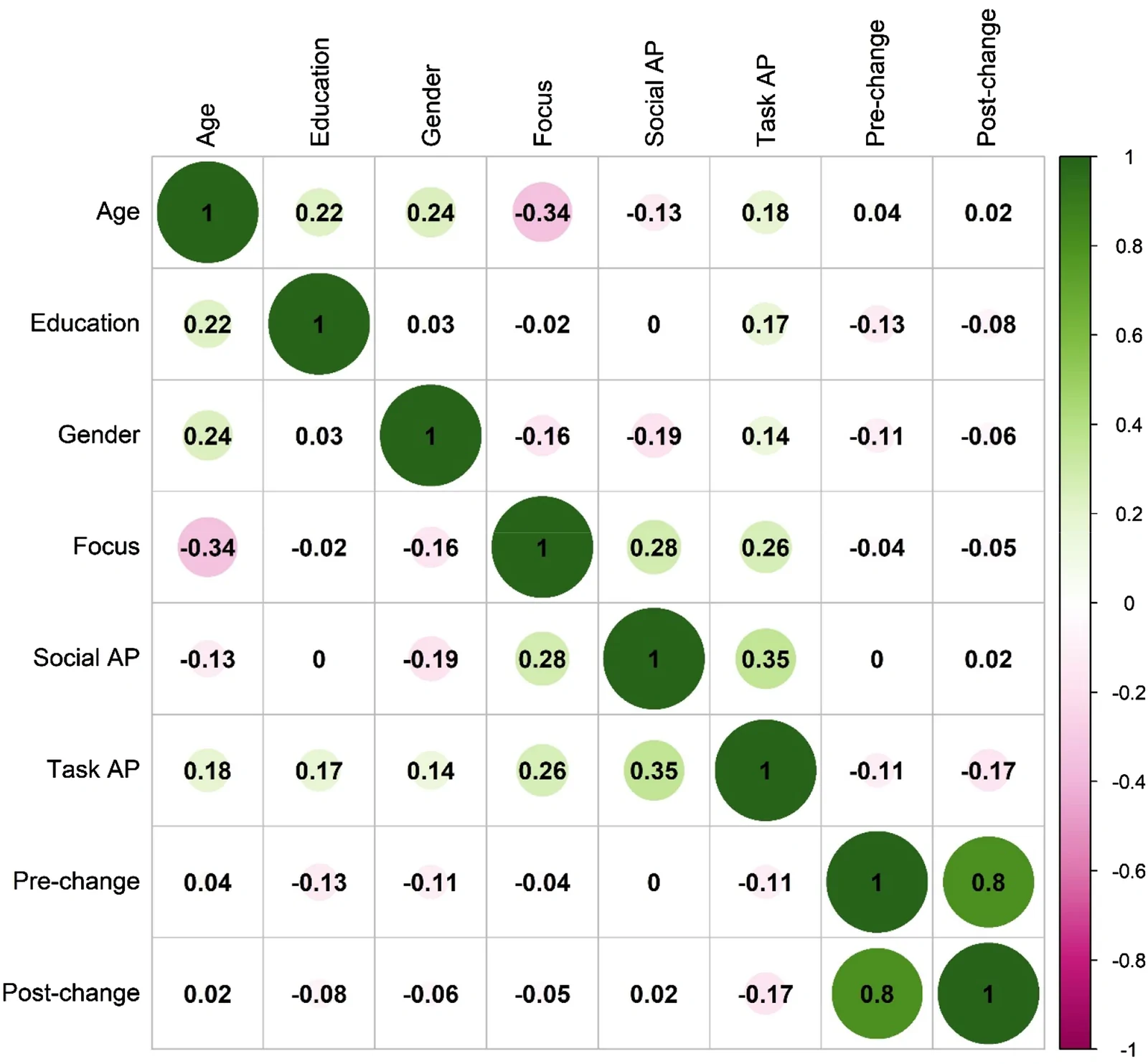
Bayesian bivariate correlations (*N* = 258) between demographic variables, focus on opportunities, subjective adaptive performance (social and task-oriented), and task performance (pre-change and post-change errors). *Note*. Gender was coded as zero = female (170) and 1 = male (88) because no people identified as intersex/diverse. Bayesian diagnostics and 95% credibility intervals are available at https://osf.io/2yeg3.

Most participants identified as female (170 women, 88 men, no intersex people). The participants’ age varied between 18 and 62 (*M* = 29.36, *SD* = 10.27). 97 people (38%) had a college degree, and most participants had jobs (173 people or 67%). Descriptive statistics for the main variables are provided in Table 2, and Figure 3 contains Bayesian bivariate correlations. Participants often reported a strong focus on opportunities and reported high adaptive performance levels. However, they generally reported slightly better social adaptive performance than task-oriented adaptive performance.

**Table 2.** Descriptive Statistics.

	*M*	*SD*	α [CR 95%]
Age	29.36	10.27	
Focus on opportunities	3.77	0.90	.84 [.81, .88]
Subjective social adaptive performance	5.73	0.83	.78 [.74, .82]
Subjective task-oriented adaptive performance	4.88	0.70	.82 [.79, .85]
Objective pre-change performance (errors)	2.38	1.31	
Objective post-change performance (errors)	1.83	1.29	

*Note. N* = 258; Focus on opportunities (1 = Not at all; 5 = Extremely); Social adaptive performance (1 = I do not agree at all, 7 = I agree completely); Task-oriented adaptive performance (1 = I do not agree at all, 7 = I agree completely); Objective performance = Mean task performance (0 = No errors, 21.75 = worst answers on all trials) before the change (blocks 1–4) and after the change (blocks 5–8); α = Reliability (Cronbach’s alpha); CR = credibility interval limits (95%).

Furthermore, the correlation between social and task-oriented adaptive performance was moderate (*r* = .35, 95% CR [.24, .45]), which confirms that it is important to distinguish between different adaptive performance dimensions.

### Focus on Opportunities and Subjective Adaptive Performance Ratings

**Figure 4.**
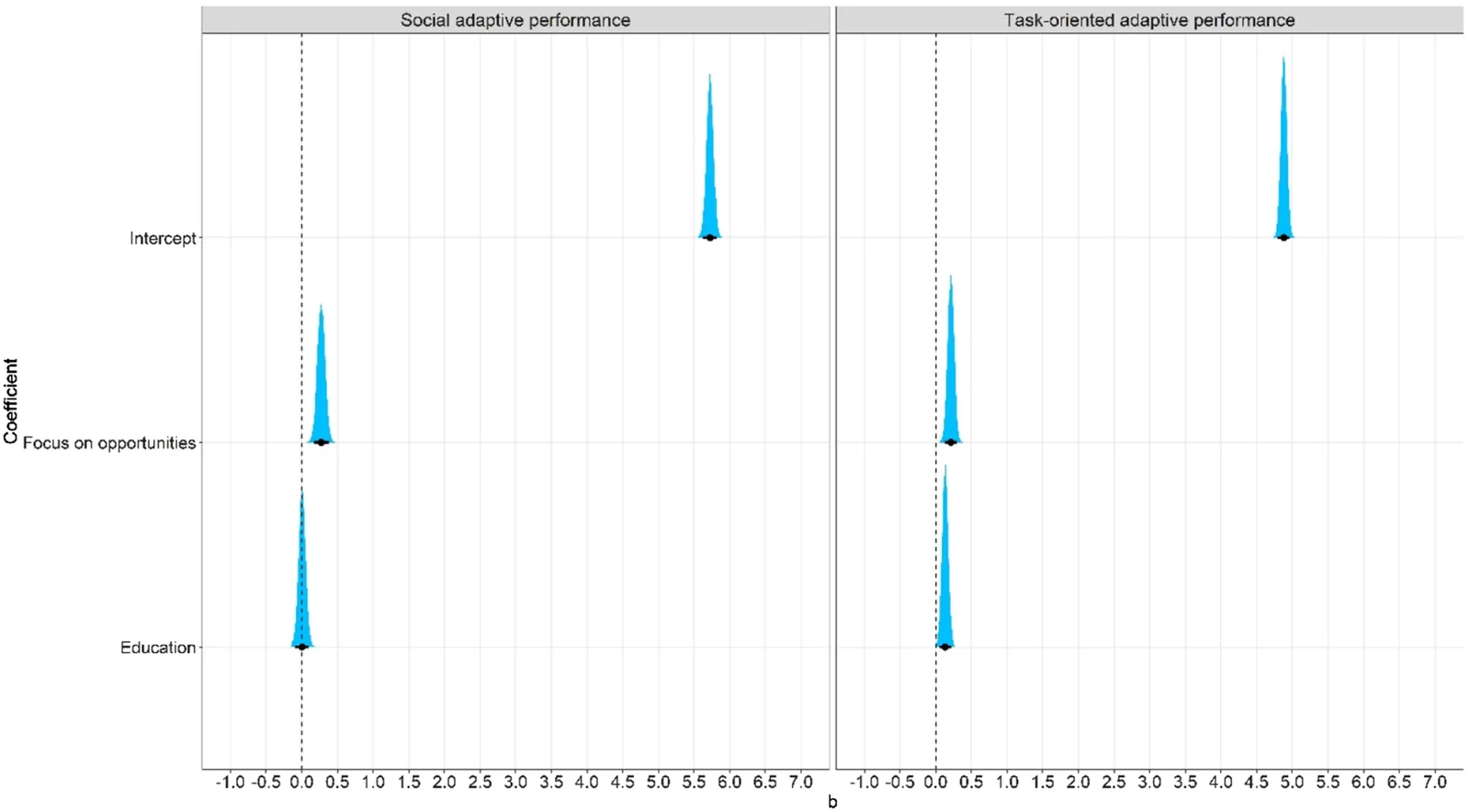
Posterior distributions for the regression models (N = 258) examining the relationship between focus on opportunities and self-reported adaptive performance (social and task-oriented) while adjusting for education. Black horizontal lines are 95% credibility intervals around the point estimates.

As expected, even after adjusting for education, focus on opportunities was related to self-reported social adaptive performance (Hypothesis 1) and self-reported task-oriented adaptive performance (Hypothesis 2). Specifically, people with a stronger focus on opportunities reported, on average, better social (*b* = 0.27, 95% CR [0.17, 0.38]) and task-oriented adaptive performance (*b* = 0.21, 95% CR [0.12, 0.30]). The posterior distributions are shown in Figure 4, and a numeric model summary is provided in Table 3.

**Table 3.** Bayesian regression Models Predicting Self-reported adaptive performance by Focus on Opportunities While Adjusting for Education as a potential Confounding variable.

	Social adaptive performance			Task-oriented adaptive performance		
	*b*	Error	CR 95%	*b*	Error	CR 95%
Intercept	5.72	0.05	[5.63, 5.82]	4.88	0.04	[4.80, 4.96]
Education	0.00	0.05	[-0.09, 0.10]	0.13	0.04	[0.05, 0.22]
Focus on opportunities	0.27	0.05	[0.17, 0.38]	0.21	0.05	[0.12, 0.30]

*Note. N* = 258; Both predictor variables (i.e., education and focus on opportunities) were centered around the mean; *b* = Regression coefficients; CR = credibility interval limits (95%); full output (e.g., chain diagnostics) is available in the supplementary materials.

### Focus on Opportunities and Objective Adaptive Performance Scores

Before examining the relationship with focus on opportunities, a model containing only change variables (basic model) was examined to ensure that the task measured adaptive performance. Figure 2 includes the average performance trajectory on the share pricing task, together with the individual trajectories of the participants. Table 4 contains the model results. The average error score at the first measurement occasion was $b_{Intercept}$ = 3.27 (CR 95% [3.10, 3.45]). As expected, the errors decreased during the pre-change phase ( $b_{SA}$ = −0.96, CR 95% [-1.09, −0.84]), with the greatest improvements being made at the beginning ( $b_{SA2}$ = 0.16, CR 95% [0.12, 0.20]). Errors increased immediately after the change ( $b_{TA}$ = 1.45, CR 95% [1.29, 1.62]), but, on average, participants could learn the modified task in the subsequent blocks. However, the post-change learning rate was lower than in the pre-change phase ( $b_{RA}$ = 0.48, CR 95% [0.32, 0.64], $b_{RA2}$ = 0.07, CR 95% [0.04, 0.10]) because even performance levels immediately after the change were already better than at the beginning of the task. Thus, there was less room for improvement. In general, participants had low error scores. Nonetheless, there was much variability between people, as indicated by the relatively large random effects (Table 4), compared to the fixed effects.

**Table 4.** Bayesian Linear Mixed-effects growth Models Predicting Change in performance (errors) as a Function of Focus on Opportunities.

Variable	Basic model			Final model			
	*b*	Error	CR 95%	*b*	Error	CR 95%
Fixed effects						
Intercept	3.27	0.09	[3.10, 3.45]	3.27	0.09	[3.10, 3.44]
Skill acquisition (SA)	−0.96	0.07	[-1.09, −0.84]	−0.13	0.1	[-0.33, 0.05]
Quadratic SA (SA^2^)	0.16	0.02	[0.12, 0.20]	−0.96	0.07	[-1.09, −0.83]
Transition adaption (TA)	1.45	0.08	[1.29, 1.62]	0.16	0.02	[0.12, 0.20]
Reacquisition adaption (RA)	0.48	0.08	[0.32, 0.64]	1.45	0.08	[1.28, 1.61]
Quadratic RA (RA^2^)	0.07	0.02	[0.04, 0.10]	0.48	0.08	[0.32, 0.64]
Education				−0.24	0.09	[-0.42, −0.07]
SA x education				0.06	0.06	[-0.06, 0.19]
SA^2^ x Education				−0.01	0.02	[-0.05, 0.03]
TA x education				−0.07	0.08	[-0.23, 0.09]
RA x education				−0.04	0.08	[-0.19, 0.12]
RA^2^ x Education				0.00	0.02	[-0.04, 0.03]
Focus				−0.13	0.10	[-0.33, 0.05]
SA x focus				0.06	0.07	[-0.08, 0.19]
SA2 x Focus				0.00	0.02	[-0.05, 0.04]
TA x focus				−0.14	0.09	[-0.32, 0.04]
RA x focus				−0.05	0.08	[-0.22, 0.11]
RA2 x Focus				0.00	0.02	[-0.04, 0.03]
Random effects (SD)						
Intercept	1.38	0.07	[1.25, 1.52]	1.35	0.07	[1.22, 1.49]
Skill acquisition (SA)	0.80	0.08	[0.65, 0.96]	0.81	0.08	[0.65, 0.96]
Quadratic SA (SA^2^)	0.24	0.03	[0.19, 0.29]	0.25	0.03	[0.19, 0.29]
Transition adaption (TA)	1.02	0.09	[0.85, 1.20]	1.02	0.09	[0.84, 1.20]
Reacquisition adaption (RA)	0.77	0.11	[0.55, 0.97]	0.77	0.11	[0.55, 0.97]
Quadratic RA (RA^2^)	0.12	0.02	[0.08, 0.16]	0.12	0.02	[0.08, 0.16]

*Note. N* = 258; Focus = Focus on opportunities; Education and focus on opportunities were centered around the mean; *b* = Regression coefficients; CR = credibility interval limits (95%); full output (e.g., correlations between random effects, chain diagnostics) is available in the supplementary materials.

**Figure 5.**
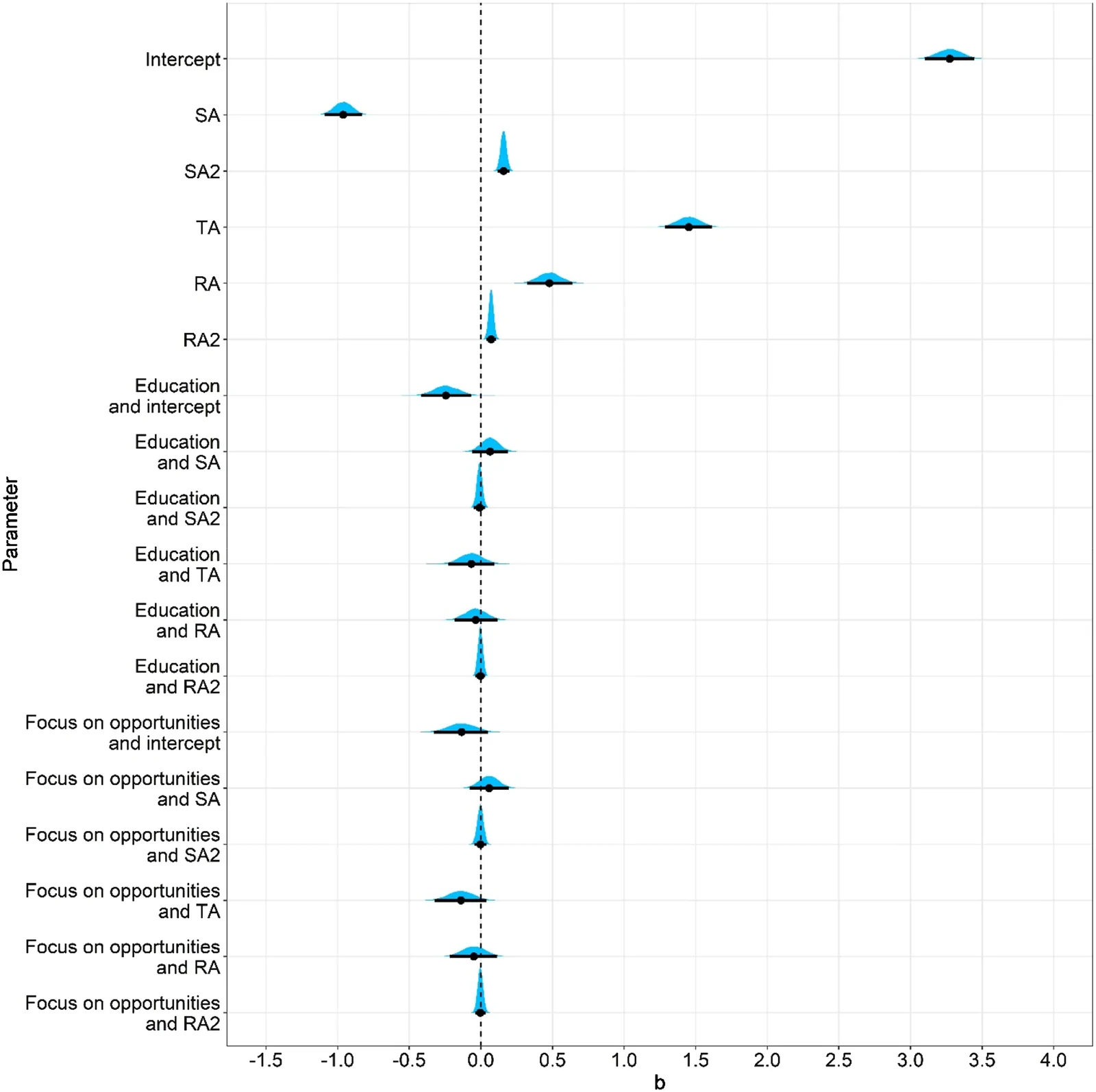
Posterior distributions for fixed effects from the model examining the relationship between focus on opportunities and performance trajectories while adjusting for education (*N* = 258). *Note*. The black horizontal lines are 95% credibility intervals around the point estimates. The posterior distributions of all interactions involving focus on opportunities contain zeros and very small values. Thus, focus on opportunities was not strongly related to performance on the task.

Next, it was examined whether trajectories differed among people with different levels of focus on opportunities. There was no substantial evidence that focus on opportunities is related to the error-based adaptive performance indicators (TA and RA) after adjusting for education. Posterior distributions are shown in Figure 5. While most values in the posterior distribution for the interaction between focus on opportunities and TA were negative (Hypothesis 3, $b_{Focus\_TA}$ = −0.14, CR 95% [-0.32, 0.04]), there were also some small positive values, which would imply that focus on opportunities is related to an increase in errors rather than facilitating transition adaption. Even more uncertainty concerning the direction of the relationship was identified for the RA parameters (Hypothesis 4). The posterior distributions contained zeros and many small values ( $b_{Focus\_RA}$ = −0.05, CR 95% [-0.22, 0.11]; $b_{Focus\_RA2}$

< 0.00, CR 95% [-0.04, 0.03]), which means that focus on opportunities does not seem to facilitate reacquisition adaption.

## Discussion

While people’s positive perceptions of their occupational future (e.g., opportunities, perceived remaining time) have been linked to better task and contextual performance (Rudolph et al., 2018), the findings are usually based on self-reported performance, which has limited validity (Heidemeier & Moser, 2009; Mabe & West, 1982). Furthermore, research examining the relationship between perceived occupational opportunities and specific performance facets was scarce. Only a few studies examined the relationship with adaptive performance, which is required in modern dynamic work environments (Abrantes et al., 2020; Shaw et al., 2019). However, both studies examined the relationship with overall future time perspective rather than specific dimensions. Research closing these research gaps and overcoming the mentioned practical limitations was required to provide nuanced recommendations about the relevance of future time perspective for organizations as well as educational institutions in personnel selection, admission tests, and employee training. Therefore, the present online study examined the relationship between adaptive performance and the dimension of future time perspective, which had the strongest relationship with other performance facets in previous studies - focus on opportunities.

Because of the limited validity of self-report performance measures and the fact that the magnitude of the relationship between predictors and adaptive performance often depends on how adaptive performance is measured (Christian et al., 2017; Stasielowicz, 2019, 2020), the present study distinguished between subjective adaptive performance ratings (i.e., questionnaire-based self-reports) and objective adaptive performance scores (i.e., errors on a task). Different patterns emerged in the current study for the respective methods. After adjusting for education as a potential confounding variable, focus on opportunities was positively related to self-reported social and task-oriented adaptive performance, but there was no substantial evidence that focus on opportunities facilitated objectively measured adaptive performance on a task. Potential explanations of this pattern and the implications for future studies are discussed in the following paragraphs.

The present finding that focus on opportunities was positively related to self-reported social and task-oriented adaptive performance aligns with meta-analytic results devoted to two broad performance facets (Rudolph et al., 2018). Specifically, Rudolph and colleagues reported a positive correlation with task and contextual performance. The raw correlation with social and task-oriented adaptive performance identified in the present study is similar (*r* = .28, 95% CR [.17, .39]; *r* = .26, 95% CR [.15, .37]) to the meta-analytic relationship with contextual performance (*r* = .24). Furthermore, it is somewhat stronger than the relationship with task performance (*r* = .10). Notably, the relationship between focus on opportunities and self-reported adaptive performance remained even after adjusting for education as a confounding variable.

However, a different pattern emerged when measuring adaptive performance more objectively (i.e., errors on a task). There was no substantial evidence that focus on opportunities facilitated adaptive performance when completing a specific task. Since objective adaptive performance indicators (transition adaption, reacquisition adaption) are based on a longitudinal framework (Bliese & Lang, 2016; Lang & Bliese, 2009), they cannot be directly compared to raw correlations. Nonetheless, it is possible to compute bivariate correlations between focus on opportunities and the average pre-change or post-change performance (i.e., errors). In the present study, there was no substantial evidence for a relationship between focus on opportunities and the average pre-change performance (i.e., task performance, *r* = −.04, 95% CR [-.16, .08]) or the post-change performance (crude measure of adaptive performance, *r* = −.05, 95% CR [-.17, .07].

While the current results based on objective adaptive performance scores appear to be less in line with the meta-analytic findings devoted to other performance facets (Rudolph et al., 2018), it is important to note that many of the studies included in the mentioned meta-analysis assessed performance via self-reports rather than objectively (Weikamp & Göritz, 2016; Zacher et al., 2010). Previous research has shown that subjective and objective performance measures (Christian et al., 2017; Conway et al., 2001; Stasielowicz, 2019, 2020) differ in their relationships with other constructs. The varying strength of the relationship confirms that subjective and objective measures cannot be used interchangeably. Although in the present study, subjective task-oriented adaptive performance ratings were related to post-change performance (errors) on the task, the relationship was only moderate (*r* = −.17, 95% CR [-.28, −.05]).

Although objective performance measures are rarely used when examining the relationship with focus on opportunities or, more generally, future time perspective, the lack of a substantial relationship between focus on opportunities and objectively measured adaptive performance is consistent with previous research. In one study that examined the relationship between the overall future time perspective and objective adaptive performance (Shaw et al., 2019), the point estimate was relatively low (*r* = .10). The authors pointed out that while future time perspective can “help with understanding causal relationships and predicting change, … it does not provide any help with generating new solutions, so the relationship may not be extremely high” (p. B-6).

Based on the described results, using focus on opportunities in personnel selection, admission tests, and employee training is currently not recommended if training or selecting for adaptability is of primary interest. On the one hand, focus on opportunities can be conceptually linked to adaptive performance through the lens of the adaption process model (Jundt & Shoss, 2023), as focus on opportunities might help anticipate or detect changes. The positive relationship identified between focus on opportunities and self-reported adaptive performance provides empirical support for this assertion. On the other hand, evidence of a relationship with objectively measured adaptive performance is needed to argue that focus on opportunities is relevant in dynamic organizational and educational contexts. While subjective measures enable one to assess several dimensions simultaneously (e.g., social and task-oriented adaptive performance), they are prone to bias when measuring performance (e.g., overestimation). In contrast, objective adaptive performance measures cannot be easily manipulated, but it usually takes more time to collect the information using tasks than questionnaires. Because of the task length, it is generally not feasible to include different tasks within a study, which would be required to ensure that these objective measures tap into multiple dimensions of adaptive performance. For this reason, the present comprehensive study cannot provide conclusive evidence about the relevance of focus on opportunities for objective adaptive performance.

### Limitations and Future Directions

While the decision to include one subjective and one objective measure of adaptive performance in the present study ensured that participant burden was acceptable, a single adaptive performance task cannot provide a definitive answer on which of the explanations of the current findings is correct. Although it is possible that focus on opportunities is generally unrelated to objective measures of adaptive performance, it cannot be ruled out that a relationship can be identified if other tasks are used. Since the current task does not involve human interaction, it cannot assess social adaptive performance. Moreover, some task-oriented dimensions are more likely to be required than others when completing the present share price forecast task. For example, when looking at the task through the lens of the original adaptive performance taxonomy (Pulakos et al., 2000), it appears that task errors in the current task reflect the dimension “learning work tasks” to a greater extent than the dimension “solving problems creatively.” Thus, one could argue that focus on opportunities might show a stronger relationship with adaptive performance on tasks tapping into other dimensions. To systematically examine this explanation, studies using several tasks are needed. One could ask participants to complete the task on different days to reduce participant burden.

It also cannot be ruled out that focus on opportunities is related to accuracy-based measures of adaptive performance to a lesser extent than to other indicators. Although researchers usually focus on accuracy when summarizing adaptive performance on a task (Stasielowicz, 2019, 2020), sometimes it might be more meaningful to use performance indicators other than errors in dynamic occupational and educational contexts. In certain dynamic situations, such as emergencies, reaction times are also critical. After all, a very accurate but slow performance can lead to deaths or monetary losses. Because of the online data collection, the reaction times could not be meaningfully compared between the participants in the present study. For example, the speed of the internet connection varies between households. However, one could consider reaction times in future laboratory studies and account for the potential speed-accuracy trade-off (Bruyer & Brysbaert, 2011; Draheim et al., 2019; Vandierendonck, 2017). Furthermore, one could use other performance indicators for simulation-based tasks. Previous examples include population increase in a city simulation (Randall et al., 2011) or eliminating opponents and surviving in a hostile simulation environment (Hardy et al., 2014).

Another explanation of the lack of a substantial relationship between focus on opportunities and objective adaptive performance in the present study is that participants are not necessarily willing to put effort into a task not given by the supervisor or personnel selection committee. Even if people with a high focus on opportunities are more likely to identify changes in a dynamic environment, they might refrain from investing resources if they think poor performance on the task will not jeopardize their future occupational opportunities. Future studies could examine this explanation by investigating the role of the perceived importance of performing a specific task well. In addition, one could examine the relevance of focus on opportunities in situations where occupational future may depend on the task outcomes, including the selection context.

Since examining all of the presented explanations for the lack of a relationship with objectively measured adaptive performance requires complex designs or conducting several independent studies in occupational and educational settings, it might be worth considering other options to provide evidence that focus on opportunities facilitates adaptive performance. Although self-reports can be a valid measure of some potential outcomes, such as job satisfaction and perceived stress, they are usually more biased when assessing performance. However, one could consider using other information sources, including supervisors, co-workers, and fellow students. Available questionnaires could be used to rate the adaptive performance of employees or peers (B. Griffin & Hesketh, 2003; Pulakos et al., 2002). While interpersonal conflicts, friendships, and lack of everyday interactions might bias the ratings, finding a relationship between focus on opportunities and adaptive performance when using multiple informants would constitute more convincing evidence than traditional self-reports.

However, researchers using subjective performance measures should ensure that the temporal causality condition is not violated (Preacher, 2015) when examining the relationship with focus on opportunities. There should be a considerable time gap between assessing focus on opportunities and performance, which could be ensured by adopting a longitudinal study design. Alternatively, researchers could modify the time frame the questionnaires are referring to. For example, focus on opportunities items could refer to the perceptions in the past months, and adaptive performance items could refer to the current week.

Finally, future studies should also consider examining focus on opportunities together with other potentially relevant variables. As the theoretical adaptive performance framework shows (Jundt & Shoss, 2023), the adaption process is a complex multi-stage process consisting of change detection, identification of change-related demands, and selecting strategies to deal with the changed environment, amongst others. Accordingly, various variables might be relevant during adaption. For example, the present study accounted for the potential influence of education, as it is related to both focus on opportunities and adaptive performance (Jundt et al., 2015; Rudolph et al., 2018). Since focus on opportunities is only one part of a self-regulatory chain, studies assessing goal setting and goal monitoring (Baird et al., 2021), in addition to focus on opportunities, could clarify the importance of the respective components. This suggestion is corroborated by the fact that learning motivation is related to both focus on opportunities and self-reported adaptive performance (Rudolph et al., 2018; Stasielowicz, 2019). Since self-regulatory processes are dynamic, it is recommended that such variables be assessed on several occasions. For example, a recent study assessed motivation before the task, before the change, and after the change (Stasielowicz, 2024). Since assessing such additional variables during the task could distract people, one could consider using process tracing methods, such as thinking aloud about the current task (Schulte-Mecklenbeck et al., 2019), which might be less invasive than questionnaires.

### Conclusion

The present study aimed to close several research gaps and overcome the limited utility of studies based exclusively on self-reported performance. Specifically, the current study examined for the first time the relationship between people’s perception of their occupational opportunities and both subjectively and objectively measured individual adaption to change. Although focus on opportunities has been previously linked to well-being and health behavior, its relationship with specific performance facets, such as adaptive performance, was largely unknown, which precluded from making specific recommendations about the use of focus on opportunities for selection and training purposes in dynamic organizations and educational institutions. To help resolve this issue, the relationship between focus on opportunities and both subjective (i.e., self-reports) and objective (i.e., task scores) adaptive performance measures was examined in the present study. Even after adjusting for education, seeing more opportunities in one’s occupational future corresponded to better self-reported social adaptive performance (e.g., dealing with new colleagues or clients from foreign cultures) and task-oriented adaptive performance (e.g., learning new procedures or dealing with emergencies). However, there was no substantial evidence that focus on opportunities facilitated performance on a specific task. Since many previous studies examining the relationship with other performance facets relied on subjective measures, it is recommended that more objective performance measures be used in future studies. Ideally, different tasks and performance indicators should be used (e.g., errors, speed, fulfilling specific criteria) to examine the discussed explanations of the present findings and ascertain whether there are circumstances under which focus on opportunities is related to objective measures of adaptive performance. Furthermore, studies that account for several self-regulatory concepts, such as focus on opportunities, goal setting, motivation, or goal monitoring, are needed to estimate the relative importance of focus on opportunities in dynamic contexts. Based on the currently available evidence, focus on opportunities should not be used in the selection context in companies and educational institutions seeking highly adaptable individuals. Relatedly, training for adaptive performance in the occupational or educational context is unlikely to benefit substantially from improving people’s perceptions of occupational opportunities.

## Data Availability

The study materials, data and analysis scripts used for this article can be accessed at https://osf.io/2yeg3/.
